# Face cooling exposes cardiac parasympathetic and sympathetic dysfunction in recently concussed college athletes

**DOI:** 10.14814/phy2.13694

**Published:** 2018-05-09

**Authors:** Blair D. Johnson, Morgan C. O'Leary, Muhamed McBryde, James R. Sackett, Zachary J. Schlader, John J. Leddy

**Affiliations:** ^1^ Center for Research and Education in Special Environments Department of Exercise and Nutrition Sciences University at Buffalo Buffalo New York; ^2^ UBMD Department of Orthopaedics and Sports Medicine University at Buffalo Buffalo New York

**Keywords:** Autonomic nervous system, blood pressure, heart rate, heart rate variability, mild traumatic brain injury, sympathetic nervous system

## Abstract

We tested the hypothesis that concussed college athletes (CA) have attenuated parasympathetic and sympathetic responses to face cooling (FC). Eleven symptomatic CA (age: 20 ± 2 years, 5 women) who were within 10 days of concussion diagnosis and 10 healthy controls (HC; age: 24 ± 4 years, 5 women) participated. During FC, a plastic bag filled with ice water (~0°C) was placed on the forehead, eyes, and cheeks for 3 min. Heart rate (ECG) and blood pressure (photoplethysmography) were averaged at baseline and every 60 sec during FC. High‐frequency (HF) power was obtained from spectral analysis of the R‐R interval. Data are presented as a change from baseline. Baseline heart rate (HC: 61 ± 12, CA: 57 ± 12 bpm; *P* = 0.69), mean arterial pressure (MAP) (HC: 94 ± 10, CA: 96 ± 13 mmHg; *P* = 0.74), and HF (HC: 2294 ± 2314, CA: 2459 ± 2058 msec^2^; *P* = 0.86) were not different between groups. Heart rate in HC decreased at 2 min (−7 ± 11 bpm; *P* = 0.02) but did not change in CA (*P* > 0.43). MAP increased at 1 min (HC: 12 ± 6, CA: 6 ± 6 mmHg), 2 min (HC: 21 ± 7, CA: 11 ± 7 mmHg), and 3 min (HC: 20 ± 6, CA: 13 ± 7 mmHg) in both groups (*P* < 0.01 for all) but the increase was greater at each interval in HC (*P* < 0.02). HF increased at 1 min (12354 ± 11489 msec^2^; *P* < 0.01) and 2 min (5832 ± 8002 msec^2^; *P* = 0.02) in HC but did not change in CA (*P* > 0.58). The increase in HF at 1 min was greater in HC versus CA (*P* < 0.01). These data indicate that symptomatic concussed patients have impaired cardiac parasympathetic and sympathetic activation.

## Introduction

Autonomic regulation of the cardiovascular system has been posited to be “uncoupled” in patients with acute traumatic brain injuries (Goldstein et al. 1998). For instance, the severity of brain injury influences autonomic function such that reductions in autonomic activity are associated with increased severity of brain injury (Lowensohn et al. 1977; Goldstein et al. 1996, 1998). In this context, concussion patients can exhibit brainstem abnormalities and lesions (Levine et al. 2008; Niogi et al. 2008; Rutgers et al. 2008; Huang et al. 2009; Polak et al. 2015), which provides structural evidence that autonomic control centers located in the brainstem could be disrupted following a concussion. Heart rate variability is an analysis technique that has been used to assess autonomic function in concussion patients and primarily gives information regarding cardiac parasympathetic activity (Joyner 2016; Michael et al. 2017). Results supporting the idea that resting autonomic function is impaired in concussion patients are inconclusive. Some investigators have found lower indices of resting heart rate variability (King et al. 1997; Hilz et al. 2011; Sung et al. 2016), whereas others have not (Gall et al. 2004; La Fountaine et al. 2009; Kozlowski et al. 2013; Abaji et al. 2016). When concussion patients have been subjected to sympathoexcitatory maneuvers, such as orthostatic challenges, exercise, and the Valsalva maneuver, heart rate variability metrics and select cardiovascular responses are altered (Gall et al. 2004; La Fountaine et al. 2009; Hilz et al. 2011, 2016; Abaji et al. 2016; Dobson et al. 2017). These findings indicate that concussion patients exhibit altered autonomic activity in response to sympathetic stimuli compared to healthy controls. Pupilometry has been used to examine parasympathetic activation in patients with postconcussion syndrome with mixed results. Concussion patients studied at least 45 days from injury exhibit impaired parasympathetic activation using pupilometry (Truong and Ciuffreda 2016). However, pupilometry was unable to detect blunted parasympathetic activation in patients tested at least 1 year from sustaining a concussion (Thiagarajan and Ciuffreda 2015). Nevertheless, it is still not known if cardiac parasympathetic activation is impaired in patients who have recently sustained a concussion.

Cooling the forehead, eyes, and cheeks (i.e., face cooling) stimulates the trigeminal nerve to evoke transient increases in cardiac parasympathetic activity (~1–2 min) and sustained increases in sympathetic activity and sympathetically mediated cardiovascular responses (Hayashi et al. 1997; Shamsuzzaman et al. 2014; Fisher et al. 2015; Schlader et al. 2016, 2018; Johnson et al. 2017). Thus, face cooling could be a novel technique to assess both branches of the autonomic nervous system in concussion patients. The purpose of our study was to examine cardiac parasympathetic activation and sympathetically mediated cardiovascular responses during face cooling in recently concussed college athletes and healthy controls. We hypothesized that recently concussed college athletes would have attenuated increases in both cardiac parasympathetic activity and sympathetically mediated cardiovascular responses during face cooling compared to healthy controls. We also explored the relationship between concussion symptoms and the autonomic responses to face cooling in the concussed athletes. We hypothesized that reported concussion symptom severity would be correlated with the autonomic responses to face cooling.

## Materials and Methods

### Participants

Eleven college athletes (5 women) who were diagnosed with a sports‐related concussion within 10 days prior to data collection and 10 healthy controls (5 women) participated in the study. Demographic data are presented in Table 1. The concussed athletes were referred to us from the University at Buffalo Concussion Management Clinic. All concussed athletes had a Glasgow Coma Scale of 15. The concussed athletes participated in National Collegiate Athletic Association Division I, II, or III sports (e.g., basketball, football, lacrosse, rowing, volleyball, and wrestling). All concussed athletes reported concussion symptoms the day of the study visit using a 22‐question Likert scale questionnaire to rate the current severity of their symptoms (None = 0, Moderate = 3, Severe = 6). The symptoms of the questionnaire included: headache, head pressure, neck pain, nausea, dizziness, blurred vision, balance problems, light sensitivity, noise sensitivity, feeling “slowed down”, feeling “in a fog”, don't feel right, difficulty concentrating, difficulty remembering, fatigue, confusion, drowsiness, trouble falling asleep, more emotional, irritability, sadness, and nervous or anxious. The sum of these responses was calculated to express a total symptom score ranging from 0 to 132. The responses were also grouped into four symptom domains: physical, cognitive, emotional, and fatigue. All concussed athletes reported that they had not sustained a previous concussion within the last year. Healthy participants reported to be recreationally active and had not sustained a concussion within the last year. A priori, we elected to not match the healthy controls to the concussed athletes for specific sport participation to avoid the potential influence of repetitive nonconcussive head impacts on autonomic function in the healthy controls (Mainwaring et al. 2018). All participants self‐reported to be free from any cardiovascular, metabolic, neurologic, respiratory, or endocrine disease. All participants also self‐reported to not be taking any medications (except birth control) and were nonsmokers. All participants provided written informed consent prior to data collection. The study was approved by the Institutional Review Board at the University at Buffalo and was performed in accordance with the standards set forth by the Declaration of Helsinki.

**Table 1:** Participant demographics

	Healthy participants	Concussed athletes	*P* value
Sex (male/female)	5/5	6/5	–
Age (years)	22 ± 2	20 ± 2	0.003
Height (cm)	175 ± 10	181 ± 10	0.206
Weight (kg)	77 ± 15	84 ± 21	0.398
BMI (kg/m^2^)	25 ± 3	25 ± 4	0.708
Days after concussion	–	5 ± 3	–

Values are means ± SD. Demographics were compared between groups using unpaired *t*‐tests.

### Instrumentation and measurements

Height and weight were measured using a stadiometer and scale (Sartorius, Bohemia, NY) at the beginning of the study visit. A 3‐lead electrocardiogram (DA100C, Biopac Systems, Goleta, CA) was used to continuously record heart rate and rhythm. Beat‐to‐beat blood pressure was collected via the Penaz method (ccNexfin, BMEYE, B.V., Amsterdam, The Netherlands or Finometer Pro, FMS, Amsterdam, The Netherlands) and intermittently confirmed using auscultation of the brachial artery via electrosphygmomanometry (Tango M2; SunTech, Raleigh, NC) and no corrections were necessary. Stroke volume was calculated using Modelflow via the blood pressure waveform (Wesseling et al. 1993).

### Experimental approach

Participants were instructed to report to the laboratory the day of the study visit after abstaining from alcohol, caffeine, and exercise for 12 h and food for 2 h. After participants were instrumented, they assumed the supine position for 10 min of quiet rest. 5 min of baseline data were acquired prior to face cooling. Face cooling was achieved by placing a pliable plastic bag filled with 2.5 L of ice water (~0°C) on the forehead, eyes, and cheeks for 3 min. Participants remained supine for 5 min following face cooling.

### Data analyses

We recorded data continuously at 1 kHz using a data acquisition system (Biopac MP150, Goleta, CA). Data were analyzed in 60 sec increments at baseline and during face cooling. Cardiac output was calculated as heart rate multiplied by stroke volume. Total peripheral resistance was calculated as mean arterial pressure divided by cardiac output. All R waves were visually inspected for ectopic beats and R‐R intervals were calculated from the ECG in 60 sec increments. Estimates of cardiac parasympathetic activity were calculated in the time domain as the root mean square of successive differences in R‐R interval (RMSSD) and high‐frequency power (HF) was calculated from the spectral analysis of the R‐R interval using WinCPRS software (Absolute Aliens, Turku, Finland). Data are presented as a change from baseline.

### Statistical analyses

Unpaired *t*‐tests were used to compare demographics and baseline values between groups. Mixed model repeated measures ANOVAs with group as a between‐subject condition and time as a within‐subject condition were used to compare cardiovascular and parasympathetic changes from baseline during face cooling. If the ANOVA revealed a significant interaction or main effect, we used the Holm‐Sidak post hoc procedure to determine where differences existed. Data over time were compared to those acquired at baseline. We also determined the nadir in heart rate and the peak increases in R‐R interval, RMSSD, HF, mean arterial pressure, systolic blood pressure, and diastolic blood pressure independent of time and compared these values between groups using unpaired *t*‐tests. Correlations between total symptom score, symptom domains (i.e., sum of physical, sum of emotional, sum of cognitive, and sum of fatigue) and the nadir heart rate and peak mean arterial pressure, RMSSD, and HF responses were made using Spearman's correlation analyses. Prism software was used to perform statistical analyses (Version 6, GraphPad Software, La Jolla, CA). Data are reported as means ± SD and statistical significance was set at *P* < 0.050.

## Results

### Heart rate, R‐R interval, RMSSD, and HF

**Figure 1:**
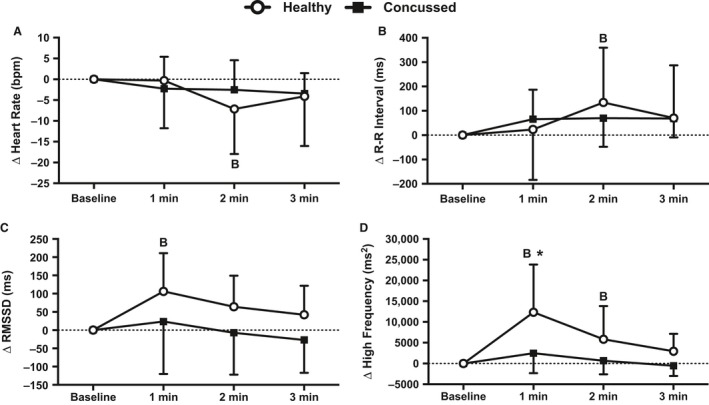
Changes in heart rate (A), R‐R interval (B), root mean square of successive differences (RMSSD) (C), and high‐frequency power (D) from baseline during 3 min of face cooling in healthy and symptomatic concussed athletes. Data were analyzed using repeated measures ANOVA followed by the Holm‐Sidak procedure for multiple comparisons if a significant interaction or main effect was found. Data are presented as mean ± SD. B = different from baseline (*P* ≤ 0.025), *different from concussed athletes (*P* < 0.001).

**Figure 2:**
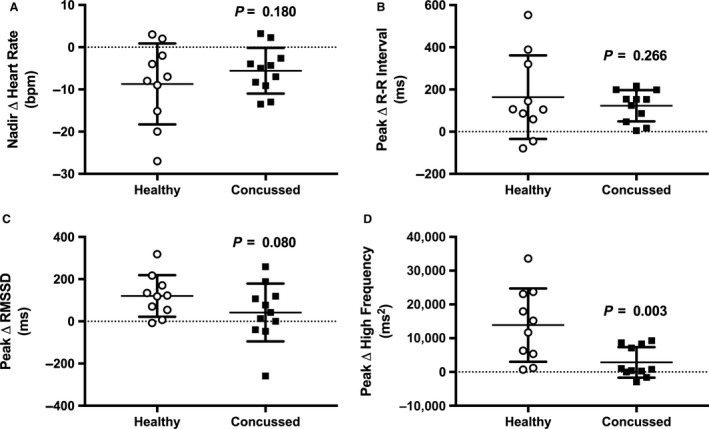
The individual responses and means ± SD for the nadir heart rate (A), and peak R‐R interval (B), root mean square of successive differences (C), and high‐frequency power (D) during face cooling independent of time. Data were analyzed using unpaired *t*‐tests.

Baseline data are presented in Table 2 and change from baseline data are presented in Figure 1. Resting heart rate (*P* = 0.687), R‐R interval (*P* = 0.877), RMSSD (*P* = 0.867), and HF (*P* = 0.864) were not different between groups at baseline. Heart rate in the concussed athletes did not change at 1 min (*P* = 0.529), 2 min (*P* = 0.529), or 3 min (*P* = 0.438) of face cooling. Heart rate in the healthy controls did not change at 1 min (*P* = 0.909), but decreased from baseline at 2 min (*P* = 0.025) of face cooling and returned to baseline at 3 min (*P* = 0.230). The change in heart rate was not different between groups at 1 min (*P* = 0.923), 2 min (*P* = 0.570), or 3 min (*P* = 0.977) of face cooling. The change in R‐R interval showed similar results compared with heart rate such that the only change was at 2 min of face cooling in the healthy controls (*P* = 0.016). RMSSD in the concussed athletes did not change from baseline at 1 min (*P* = 0.703), 2 min (*P* = 0.792), or 3 min (*P* = 0.703) of face cooling. RMSSD in the healthy controls increased at 1 min of face cooling (*P* = 0.002) and returned to baseline at 2 min (*P* = 0.058) and 3 min (*P* = 0.150). Despite the large increase in RMSSD at 1 min of face cooling in the healthy controls, there were no statistical differences between groups at 1 min (*P* = 0.158), 2 min (*P* = 0.217), or 3 min (*P* = 0.217) of face cooling. HF in the concussed athletes was not different than baseline at 1 min (*P* = 0.583), 2 min (*P* = 0.938), or 3 min (*P* = 0.938) of face cooling. HF in the healthy controls significantly increased at 1 min (*P* < 0.001) and 2 min (*P* = 0.024) and returned to baseline at 3 min (*P* = 0.197) of face cooling. The increase in HF in the healthy controls was greater than the concussed athletes at 1 min (*P* < 0.001) but was not different at 2 min (*P* = 0.105) and 3 min (*P* = 0.282) of face cooling. Figure 2 displays the individual responses and means ± SD for the greatest change from baseline, independent of time, in heart rate, R‐R interval, RMSSD, and HF during face cooling. The nadir in heart rate (*P* = 0.180) and increases in R‐R interval (*P* = 0.266) and RMSSD (*P* = 0.080) were not different between concussed athletes and healthy controls during face cooling. Concussed athletes had attenuated increases in HF versus healthy controls during face cooling (*P* = 0.003).

**Table 2:** Baselines cardiovascular values

	Healthy participants	Concussed athletes	*P* value
Heart rate (bpm)	61 ± 12	57 ± 12	0.689
R‐R interval (msec)	1022 ± 197	1009 ± 168	0.877
RMSSD (msec)	101 ± 83	108 ± 99	0.867
High frequency (msec^2^)	2294 ± 2314	2459 ± 2058	0.864
Stroke volume (mL)	106 ± 24	105 ± 27	0.902
Cardiac output (L/min)	6.1 ± 1.6	6.3 ± 1.6	0.768
Mean arterial pressure (mmHg)	94 ± 10	96 ± 13	0.737
Systolic blood pressure (mmHg)	129 ± 16	135 ± 16	0.407
Diastolic blood pressure (mmHg)	74 ± 8	74 ± 11	0.876
Total peripheral resistance (mmHg/L per min)	16.4 ± 7.4	16.5 ± 4.3	0.981

Values are means ± SD. RMSSD = root mean square of successive differences in R‐R interval. Baseline values were compared between groups using unpaired *t*‐tests.

### Stroke volume and cardiac output

**Figure 3:**
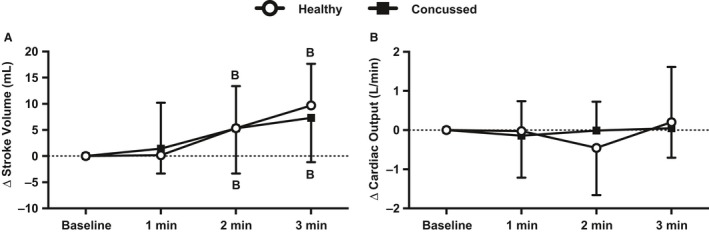
Changes in stroke volume (A) and cardiac output (B) from baseline during 3 min of face cooling in healthy and symptomatic concussed athletes. Data were analyzed using repeated measures ANOVA followed by the Holm‐Sidak procedure for multiple comparisons if a significant interaction or main effect was found. Data are presented as mean ± SD. B, different from baseline (*P* ≤ 0.044).

Baseline data are presented in Table 2 and change from baseline data are illustrated in Figure 3. Resting stroke volume (*P* = 0.902) and cardiac output (*P* = 0.768) were not different between groups. Stroke volume in the concussed athletes did not change at 1 min of face cooling (*P* = 0.510) but was greater than baseline at 2 min (*P* = 0.035) and 3 min (*P* = 0.004) of face cooling. Stroke volume in the healthy controls was also not changed at 1 min of face cooling (*P* = 0.944) but was greater than baseline at 2 min (*P* = 0.044) and 3 min (*P* < 0.001) of face cooling. Stroke volume was not different between groups at any face cooling interval (*P* > 0.902 for all). The change in cardiac output was not different throughout face cooling (*P* > 0.421) or between groups (*P* > 0.886).

### Blood pressure and total peripheral resistance

**Figure 4:**
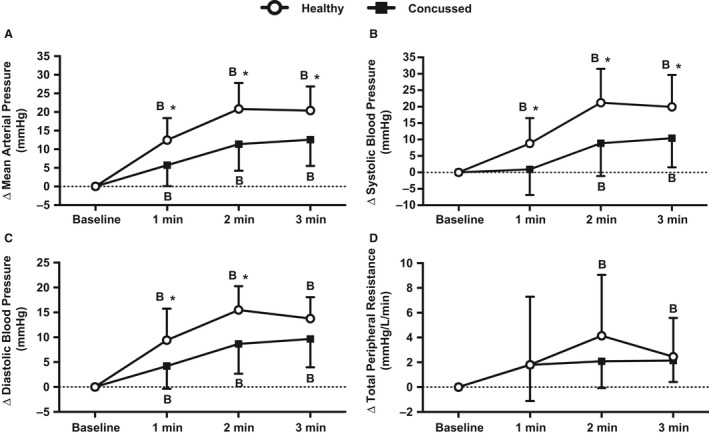
Changes in mean arterial pressure (A), systolic blood pressure (B), diastolic blood pressure (C), and total peripheral resistance (D) from baseline during 3 min of face cooling in healthy and symptomatic concussed athletes. Data were analyzed using repeated measures ANOVA followed by the Holm‐Sidak procedure for multiple comparisons if a significant interaction or main effect was found. Data are presented as mean ± SD. B = different from baseline (*P* ≤ 0.032), *different from concussed athletes (*P* ≤ 0.049).

**Figure 5:**
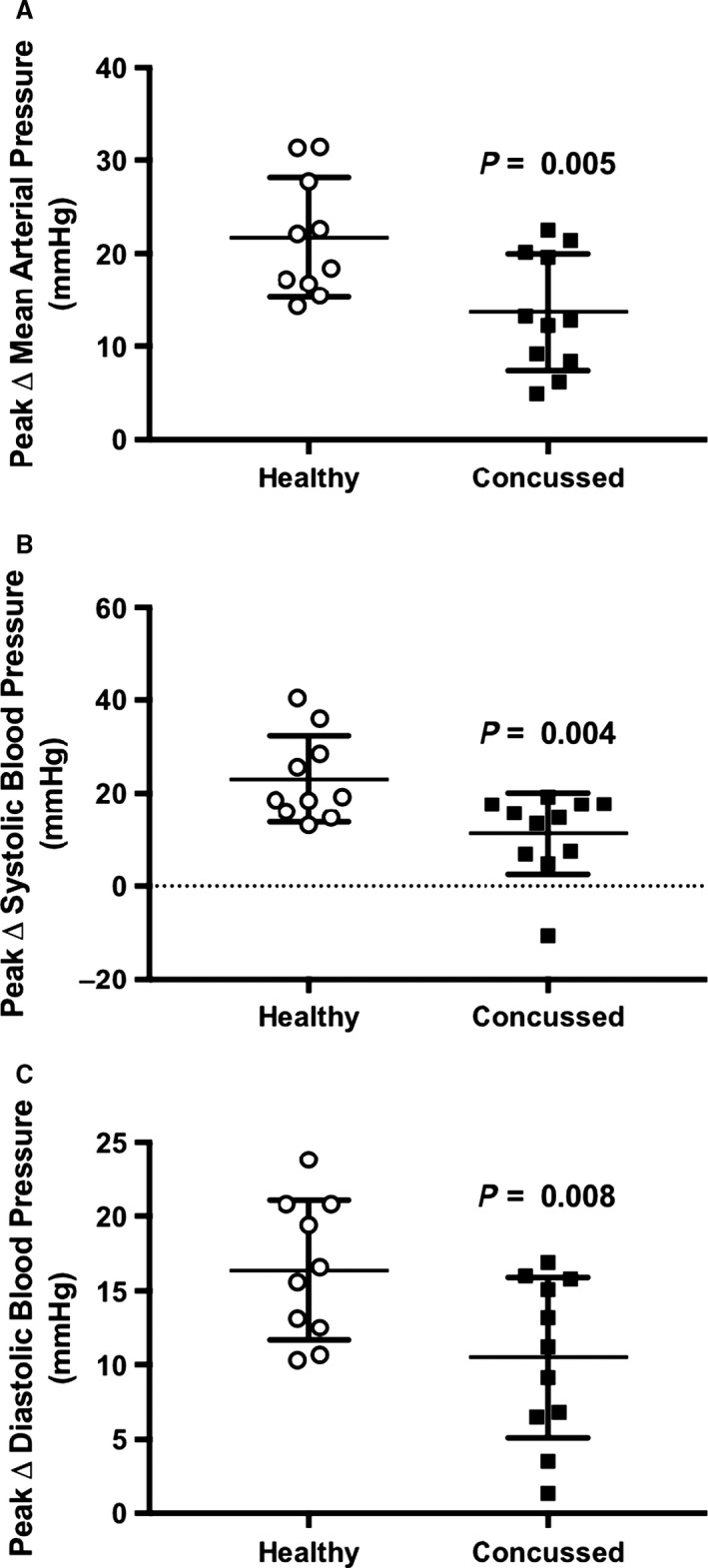
The individual responses and means ± SD for the peak increases in mean arterial pressure (A), systolic blood pressure (B), and diastolic blood pressure (C) during face cooling independent of time. Data were analyzed using unpaired *t*‐tests.

Baseline data are presented in Table 2 and change from baseline data are illustrated in Figure 4. Resting mean arterial pressure (*P* = 0.737), systolic blood pressure (*P* = 0.407), diastolic blood pressure (*P* = 0.876), and total peripheral resistance (0.981) were not different between groups. Mean arterial pressure in the concussed athletes significantly increased at 1 min (*P* = 0.003), 2 min (*P* < 0.001), and 3 min (*P* < 0.001) of face cooling. Mean arterial pressure in the healthy controls was also increased at 1 min (*P* < 0.001), 2 min (*P* < 0.001), and 3 min (*P* < 0.001) of face cooling. The increase in mean arterial pressure was greater in the healthy controls versus the concussed athletes at 1 min (*P* = 0.016), 2 min (*P* = 0.001), and 3 min (*P* = 0.007) of face cooling. Systolic blood pressure in the concussed athletes was not different from baseline at 1 min (*P* = 0.738) of face cooling but was greater at 2 min (*P* = 0.003) and 3 min (*P* < 0.001). Systolic blood pressure in the healthy controls was greater than baseline at 1 min (*P* = 0.002), 2 min (*P* < 0.001), and 3 min (*P* < 0.001) of face cooling. The increase in systolic blood pressure was greater in the healthy controls vs. the concussed athletes at 1 min (*P* = 0.049), 2 min (*P* = 0.003), and 3 min (*P* = 0.022) of face cooling. Diastolic blood pressure in the concussed athletes was greater than baseline at 1 min (*P* = 0.009), 2 min (*P* < 0.001), and 3 min (*P* < 0.001) of face cooling. Diastolic blood pressure in the healthy controls was greater than baseline at 1 min (*P* < 0.001), 2 min (*P* < 0.001), and 3 min (*P* < 0.001) of face cooling. The increase in diastolic blood pressure was greater in the healthy controls vs. the concussed athletes at 1 min (*P* = 0.035) and 2 min (*P* = 0.005) but not at 3 min (*P* = 0.089) of face cooling. Total peripheral resistance in the concussed athletes was not different at 1 min (*P* = 0.077), 2 min (*P* = 0.077), or 3 min (*P* = 0.077) of face cooling. Total peripheral resistance in the healthy controls was not different at 1 min (*P* = 0.073) but was greater than baseline at 2 min (*P* < 0.001) and 3 min (*P* = 0.032) of face cooling. The change in total peripheral resistance was not different between groups at 1 min (*P* > 0.999), 2 min (*P* = 0.436) or 3 min (*P* = 0.994) of face cooling. Figure 5 displays the individual responses and means ± SD for the greatest change from baseline, independent of time, in mean arterial pressure, systolic blood pressure, and diastolic blood pressure during face cooling. Concussed athletes had attenuated increases in mean arterial pressure (*P* = 0.005), systolic blood pressure (*P* = 0.004), and diastolic blood pressure (*P* = 0.008) versus healthy controls.

### Correlation between total symptom score and symptom domains and heart rate, mean arterial pressure, RMSSD, and HF responses during face cooling

Total symptom score and individual symptom domain scores are reported in Table 3. Spearman's correlation coefficients are reported in Table 4. There were no significant correlations between symptoms or autonomic responses during face cooling (all *P* > 0.079).

**Table 3:** Reported concussion symptom scores

Total symptom score (0–132)	37 ± 25
Physical (0–54)	14 ± 10
Cognitive (0–36)	13 ± 8
Emotional (0–24)	4 ± 4
Fatigue (0–18)	4 ± 3

**Table 4:** Spearman's correlation (*r*_s_) between concussion symptoms and autonomic responses during face cooling

	Nadir heart rate	Peak MAP	Peak RMSSD	Peak high frequency
Total symptom score	0.34	−0.20	−0.10	0.25
Physical	0.30	−0.19	−0.34	0.08
Cognitive	0.25	−0.26	−0.11	0.34
Emotional	0.56	−0.20	0.11	0.02
Fatigue	0.16	−0.23	−0.17	0.18

Values are *r*_*s*_. MAP, mean arterial pressure; RMSSD, root mean square of successive differences in R‐R interval. All correlations are *P* > 0.079.

## Discussion

The primary novel finding from our study is that recently concussed college athletes do not exhibit the typical increases in RMSSD and HF that occur in healthy controls during face cooling. Furthermore, the concussed athletes had impaired pressor responses when compared to healthy controls during face cooling. Collectively, our data indicate that recently concussed college athletes have impaired activation of both the parasympathetic and sympathetic branches of the autonomic nervous system.

We found that face cooling‐induced increases in cardiac parasympathetic activity (e.g., RMSSD and HF) were virtually absent in recently concussed college athletes. Along these lines, average pupil constriction velocity in response to a brief visual light stimulus is also an indicator of parasympathetic activation (Truong and Ciuffreda 2016). Concussion patients more than 45 days from injury exhibit attenuated average pupil constriction velocity (Truong and Ciuffreda 2016) indicating an attenuated ability to activate the parasympathetic nervous system, which agree with our results. However, symptomatic concussion patients who were more than a year from injury did not show impairments in pupillary dynamics in response to visual light stimuli (Thiagarajan and Ciuffreda 2015). It is possible that the ability to activate the parasympathetic nervous system returns to normal over time even if patients are still symptomatic. However, we did not re‐assess our concussed patients to determine if cardiac parasympathetic responses during face cooling revert to healthy control values. Further research is needed to determine if cardiac parasympathetic activation returns to healthy control values when concussion patients are asymptomatic and have resumed normal activities.

Some investigations have assessed heart rate variability in concussed patients during various sympathoexcitatory maneuvers. However, deducing which branch of the autonomic nervous system is abnormal in concussion patients during sympathoexcitatory maneuvers using heart rate variability metrics is difficult because heart rate variability metrics largely represent parasympathetic activity (Joyner 2016; Michael et al. 2017). Nonetheless, some heart rate variability metrics, such as RMSSD and HF, have been found to be abnormal in concussion patients during standing (Hilz et al. 2011), isometric handgrip exercise (Abaji et al. 2016), and submaximal cycle ergometer aerobic exercise (Gall et al. 2004). Thus, these previous investigations do provide evidence of autonomic dysfunction but it is not clear if these abnormalities are due to heightened sympathetic activation or parasympathetic withdrawal. Unlike these earlier studies where heart rate variability should be expected to decrease during sympathoexcitation, face cooling initially and transiently increases cardiac parasympathetic activity (Hayashi et al. 1997; Fisher et al. 2015; Schlader et al. 2016; Johnson et al. 2017) followed by increased sympathetic activity (Shamsuzzaman et al. 2014; Fisher et al. 2015). The infusion of atropine, a muscarinic receptor antagonist, attenuates the decrease in heart rate during face cooling (Heistad et al. 1968; Finley et al. 1979). In this context, cardiac parasympathetic dysfunction in patients with familial dysautonomia has been shown using cold stimulation of the trigeminal nerve (Hilz et al. 1999). Furthermore, the infusion of propranolol, a nonselective beta adrenergic receptor blocker, blunts the rise in blood pressure during face cooling (Finley et al. 1979). Therefore, face cooling appears to be a suitable approach to discern which branch of the autonomic nervous system is altered in concussion patients.

We found that concussed athletes had only 50% of the increase in mean arterial pressure that was exhibited in the healthy controls. Because we did not find any changes in cardiac output during face cooling or differences between groups, it appears as though most of the differential pressor responses between groups are due to lack of changes in total peripheral resistance in the concussed athletes. These data suggest that sympathetic activation and/or vascular responsiveness to neural activation is blunted in the concussed athletes during face cooling. We speculate that the primary cause of the lack of an increase in total peripheral resistance is due to a reduction in sympathetic outflow in the concussed athletes, possibly owing to brainstem abnormalities (Levine et al. 2008; Niogi et al. 2008; Rutgers et al. 2008; Huang et al. 2009; Polak et al. 2015). The return to resting systolic blood pressure after a Valsalva maneuver has been shown to be delayed in concussion patients (Hilz et al. 2016; Dobson et al. 2017). Contrary to our results, these findings suggest persistent sympathetic activation in concussion patients. However, concussion patients have shown greater reductions in systolic blood pressure during orthostatic tests (Dobson et al. 2017), which are in line with our results indicating attenuated sympathetic activation in concussed athletes. Furthermore, concussed patients demonstrate reduced resting cardiac baroreflex sensitivity (Hilz et al. 2011, 2016) that might predispose them to larger reductions in blood pressure during orthostatic tests.

Contrary to our hypothesis, we did not find any significant correlations between reported symptoms and autonomic responses during face cooling in the concussed athletes. Although further research is warranted, these data suggest that there is a disconnect between symptoms and autonomic activation. Consequently, concussed athletes might report a low number of symptoms but still not be physiologically recovered. This underscores the need to establish objective biomarkers of concussion to lower the occurrence of allowing athletes to return to play before they are physiologically recovered.

### Experimental considerations

Our study has several limitations that are worth noting. First, our healthy controls were slightly older than the concussed athletes. However, it is unlikely that the small difference in age significantly impacted the physiological responses to face cooling in our study. Second, we did not match the concussed athletes to healthy controls for athletic activity. However, the healthy controls reported to be recreationally active and this approach allowed us to avoid any potential influence that repeated nonconcussive head impacts might have on the face cooling responses by not using healthy athletes as controls. In this context, we did not track activity of the concussed athletes after they sustained their injury. Our clinic typically recommends that concussed patients perform light‐moderate physical activity within 2–3 days following the concussive injury and avoid “cocoon therapy”. Therefore, it is unlikely that a few days of limited physical activity influenced our results. Third, we did not assess perceived pain during the face cooling stimulus. Although unlikely, it is possible that the concussed athletes had a greater pain tolerance during face cooling. Fourth, we did not assess cardiac or arterial function. A reduction in cardiac and/or arterial function might have influenced responsiveness to neural stimulation during face cooling. Although we cannot rule out cardiac or arterial dysfunction, it is unlikely that any changes in cardiac or arterial function due to a reduction in physical activity would contribute to the differences in the autonomic and cardiovascular responses that we observed during face cooling. Along these lines, neural recordings of sympathetic nerve activity would give us direct insight whether sympathetic responses were different between groups without relying on end organ responses. In this regard, several limitations of using heart rate variability during the face cooling procedure should be noted. Typically, indices of heart rate variability are assessed in durations greater than 5 min, whereas assessed RMSSD and HF in relatively short segments, as we (Schlader et al. 2016, 2018; Johnson et al. 2017) and others (Hilz et al. 1999; Rickards et al. 2010; Fisher et al. 2015) have done previously. Along these lines, we calculated HF during periods where autonomic responses were shifting from parasympathetic activation to sympathetic activation and fast Fourier transformation relies on a relatively stable signal for proper analysis. However, we are confident in our HF data because these responses are similar to the RMSSD responses. We also did not measure or control respiratory rate during face cooling. Cold stimulation of the trigeminal nerve can induce apneas (Pendergast et al. 2015) which could influence heart rate variability (Camm et al. 1996). Finally, we did not determine if cerebral abnormalities were present in either group using neuroimaging techniques. This might have provided us with structural information about whether or not autonomic cardiovascular centers located in the brainstem or insula were abnormal and could have contributed to our results.

### Perspectives and significance

Diagnosis of a concussion can be difficult and is often reliant on subjective self‐reporting of concussion symptoms. Furthermore, sustaining a head injury before the brain has sufficiently healed can prolong recovery and worsen symptoms (Longhi et al. 2005; Tavazzi et al. 2007; Vagnozzi et al. 2008, 2010). Therefore, identifying an objective biomarker of concussion based on physiological responses to a stressor could improve the initial diagnosis of concussion and influence decisions regarding return to activity. Several blood tests have received interest as objective biomarkers of concussion (Jeter et al. 2013). However, a major problem with blood biomarkers is that it may take several days before results are known and they involve complex assays, which might not be feasible. Exercise stress tests have been used to determine exercise tolerance in patients to assist with concussion diagnosis (Leddy et al. 2010, 2011; Kozlowski et al. 2013; Darling et al. 2014; Clausen et al. 2015) but many concussion clinics do not have the necessary personnel and equipment required to safely perform graded exercise stress tests. On the other hand, face cooling is relatively straightforward, quick, and does not rely on patient effort. Therefore, face cooling could be used with other standard diagnostic tools and techniques to aid in concussion diagnosis. However, the specificity and sensitivity of face cooling to diagnose concussion and to establish physiological recovery warrants further investigation.

## Conclusions

We found that recently concussed college athletes exhibit impaired cardiac parasympathetic and sympathetic activation during face cooling compared to healthy controls. These data indicate that both branches of the autonomic nervous system are dysfunctional following a concussion.

## Conflict of Interest

There are no conflicts of interest to report.
